# Quantification of Protein Copy Number in Yeast: The NAD^+^ Metabolome

**DOI:** 10.1371/journal.pone.0106496

**Published:** 2014-09-04

**Authors:** Szu-Chieh Mei, Charles Brenner

**Affiliations:** Department of Biochemistry, Carver College of Medicine, University of Iowa, Iowa City, Iowa, United States of America; Texas A&M University, United States of America

## Abstract

*Saccharomyces cerevisiae* is calorie-restricted by lowering glucose from 2% to 0.5%. Under low glucose conditions, replicative lifespan is extended in a manner that depends on the NAD^+^-dependent protein lysine deacetylase Sir2 and NAD^+^ salvage enzymes. Because NAD^+^ is required for glucose utilization and Sir2 function, it was postulated that glucose levels alter the levels of NAD^+^ metabolites that tune Sir2 function. Though NAD^+^ precursor vitamins, which increase the levels of all NAD^+^ metabolites, can extend yeast replicative lifespan, glucose restriction does not significantly change the levels or ratios of intracellular NAD^+^ metabolites. To test whether glucose restriction affects protein copy numbers, we developed a technology that combines the measurement of Urh1 specific activity and quantification of relative expression between Urh1 and any other protein. The technology was applied to obtain the protein copy numbers of enzymes involved in NAD^+^ metabolism in rich and synthetic yeast media. Our data indicated that Sir2 and Pnc1, two enzymes that sequentially convert NAD^+^ to nicotinamide and then to nicotinic acid, are up-regulated by glucose restriction in rich media, and that Pnc1 alone is up-regulated in synthetic media while levels of all other enzymes are unchanged. These data suggest that production or export of nicotinic acid might be a connection between NAD^+^ and calorie restriction-mediated lifespan extension in yeast.

## Introduction

Calorie restriction (CR) is a powerful intervention to extend lifespan and healthspan in model organisms including yeast, flies, worms and rodents [1], [2]. Although recent studies on the efficacy of CR to extend lifespan in primates were equivocal, salutary effects on biochemical parameters were observed in both studies [3], [4]. Thus, the molecular mechanism by which CR promotes healthful changes remains of interest.

*Saccharomyces cerevisiae* is considered an excellent tool to study lifespan because it is a single-celled eukaryote with a rapid cell division cycle, compact genome, and unparalleled genetic control. In yeast, simply lowering the glucose concentration from 2% to 0.5% produces CR [5], [6]. Previous studies have shown that replicative lifespan (RLS) can be extended by CR in a manner that depends on Sir2 [5], a nicotinamide adenine dinucleotide (NAD^+^)-dependent protein lysine deacetylase [7], [8], and genes encoding NAD^+^ salvage enzymes [9]. Because NAD^+^ is required for glucose fermentation and for Sir2 function, it was postulated that levels of glucose alter aspects of NAD^+^ metabolism to produce a signal that increases Sir2 activity, thereby extending lifespan [10], [11]. Two competing models were put forth: that CR would increase the NAD^+^: NADH ratio [12], or that CR would increase the NAD^+^: nicotinamide (Nam) ratio [13]. Both models posit that NADH or Nam, proposed as inhibitory metabolites, achieve levels in cells that would inhibit Sir2 activity.

Despite classical analysis of NAD^+^ metabolism in the yeast system [14], [15], the set of genes, enzymes, transporters and metabolites was incomplete at the time both models were proposed [12], [13]. Surprisingly, we found that nicotinamide riboside (NR), a natural product found in milk, is capable of bypassing all known pathways to NAD^+^ by virtue of the activity of a specific NR kinase pathway [16]. We further expanded the NAD^+^ metabolome with biochemical and genetic characterization of NAD^+^ biosynthetic enzymes [17]–[23]. Once all genes for salvage of NR to NAD^+^ were identified, we showed that addition of NR to synthetic high glucose media increased Sir2 activity and extended lifespan so long as the NR salvage genes were present and extended lifespan correlated with the ability of NR to elevate NAD^+^ [20]. Though these data indicate that an intervention that increases NAD^+^ extends lifespan, it does not necessarily follow that the mechanism by which CR works is increasing NAD^+^ or altering ratios of NAD^+^ metabolites. We therefore developed LC-MS methods to quantify the expanded NAD^+^ metabolome [24], [25] and used these methods to determine what happens to NAD^+^ metabolites under two conditions that extend lifespan, namely provision of NAD^+^ precursors and glucose restriction.

Our data indicate that intracellular NAD^+^ metabolite levels range from below 0.1 µM nicotinic acid adenine dinucleotide (NAAD) to above 500 µM (NAD^+^) in yeast cells. Inclusion of nicotinic acid (NA) or yeast extract in media increases intracellular NAD^+^ levels and increases concentrations of all NAD^+^ metabolites including NADH and Nam [24]. These data are consistent with our previous observation that NR supplementation increases yeast intracellular NAD^+^ levels and extends lifespan [20] and they challenge the view that NADH [26] or Nam are inhibitory metabolites.

Though intracellular NAD^+^ metabolites were not altered by glucose restriction, we aimed to determine whether the abundance of NAD^+^ biosynthetic proteins is altered consistent with predictions that the flux of NAD^+^ metabolism may be increased by CR [13]. To answer this question, we developed a quantitative method to determine the copy number of molecules in the yeast proteome and discovered that copy numbers of NAD^+^ enzymes range from 300 to 49,000 per diploid cell. Our data indicate that the levels of most NAD^+^ metabolic enzymes are not altered. However, in rich media, Sir2, an enzyme that produces Nam from NAD^+^, and Pnc1, the enzyme that hydrolyzes Nam to NA, are up-regulated in glucose-restricted conditions, while only Pnc1 is up-regulated in glucose-restricted synthetic media conditions. These data suggest that NA metabolism may be functionally modulated by glucose restriction in a manner that promotes increased lifespan.

## Materials and Methods

### Strains and media

**Figure 1:**
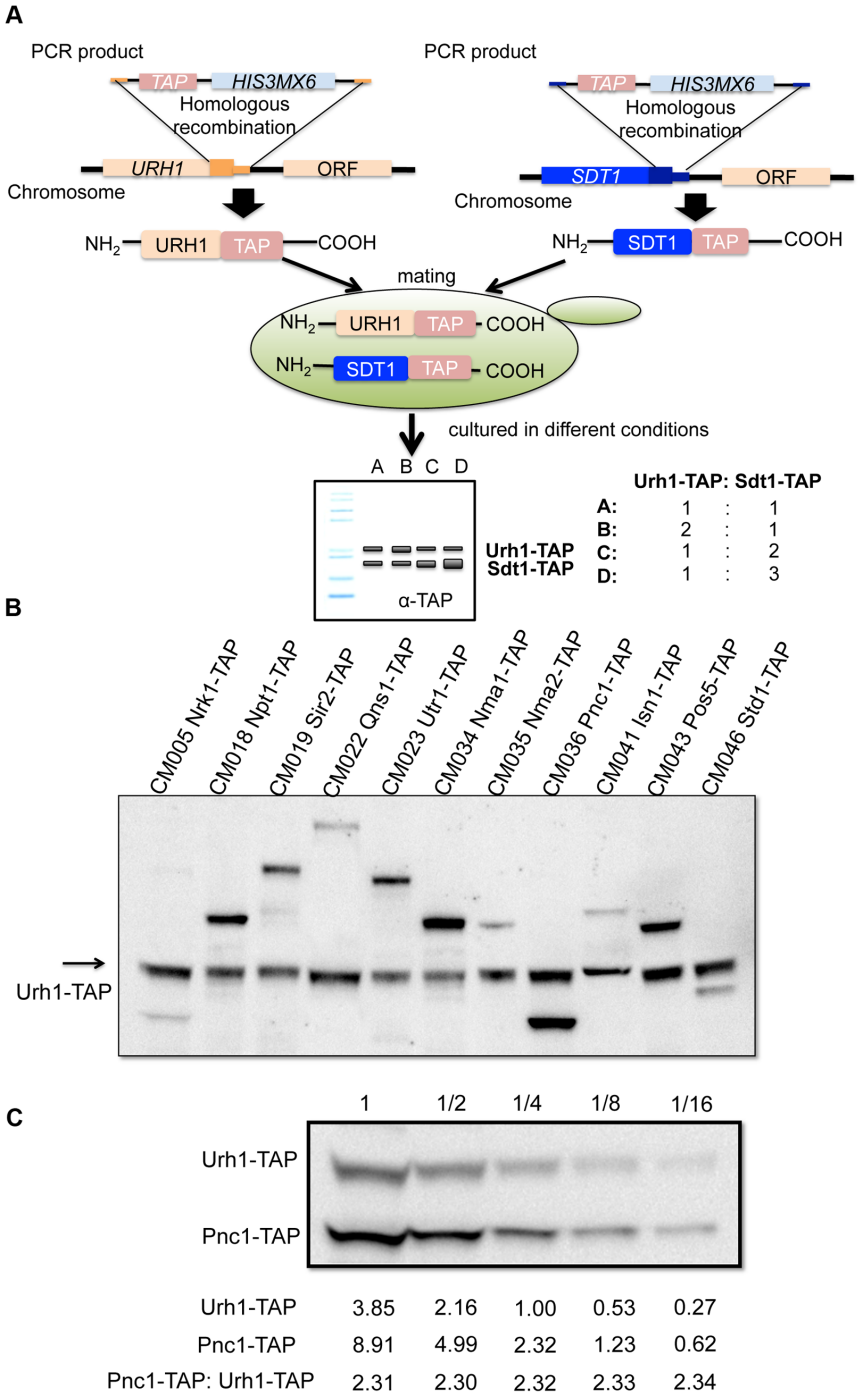
Dually TAP-tagged yeast strains to determine copy number relative to Urh1. (A) Strain construction to generate dually TAP-tagged strains, permitting relative protein quantification by western blot. (B) Dually tap-tagged strains at 2% glucose establish the specificity of the western assay. (C) Cell extracts from CM036 (Urh1-TAP, Pnc1-TAP) analyzed over a range of dilutions establish the linearity of TAP-tagged detection.

Yeast strains used in this study are listed in Table 1. BY4741 and TAP-tagged strains except those encoding Sdt1-TAP were purchased from Open Biosystems. CM044 was obtained by genomic integration of the TAP tag as a C-terminal fusion as shown in Figure 1A. In brief, a TAP tag fragment flanked with 3′ *SDT1* sequences was first amplified from genomic DNA of PAB054 (Urh1-TAP) with primers Sdt1-TAPF (5′- TGATATATTGGAGTTACCACACGTTGTGTCCGACCTGTTCGGTCGACGGATCCCCGGGTT-3′) and Sdt1-TAPR (5′- ATAGAGGCATCTAATGCAAGTAGATTTATATACAATTATATCGATGAATTCGAGCTCGTT-3′). The PCR product was than transformed into BY4741 cells and plated on SDC-His media for selection. CM044 was obtained after diagnostic PCR with primers Sdt1F (5′- GACTACTCTAGGACAGATAC-3′) and Sdt1R (5′- CTAACTGCTATGATCATCAG-3′) demonstrated a 2219 bp product. CM003, a MATα Urh1-TAP strain, was obtained by introducing a *GAL1-HO* plasmid to PB054 and passage through galactose media to induce a mating type switch. To obtain dually tagged diploid yeast strains, all MAT**a**, *i.e.* BY4741-derived TAP-tag strains were crossed with CM003. Media used for protein copy number analysis was YP (2% bacto peptone, 1% yeast extract) or synthetic complete media supplemented with filter-sterilized glucose at final concentrations of 2%, 0.5% or 0.2%.

**Table 1:** Yeast strains used in this study.

Strain	Genotype
BY4741	*MATa his3* Δ1 *leu*2Δ0 *lys*2Δ0 *ura*3Δ0
CM001	*MAT*a *urh1*Δ::KanMX *his*3Δ1 *leu*2Δ0 *lys*2Δ0 *ura*3Δ0
PAB030	BY4741 NRK1::TAP-HIS3MX6
PAB054	BY4741 URH1::TAP-HIS3MX6
CM006	BY4741 NPT1::TAP-HIS3MX6
CM007	BY4741 SIR2::TAP-HIS3MX6
CM010	BY4741 QNS1::TAP-HIS3MX6
CM011	BY4741 UTR1::TAP-HIS3MX6
CM024	BY4741 NMA1::TAP-HIS3MX6
CM025	BY4741 NMA2::TAP-HIS3MX6
CM026	BY4741 PNC1::TAP-HIS3MX6
KB048	BY4741 ISN1::TAP-HIS3MX6
CM037	BY4741 POS5::TAP-HIS3MX6
CM044	BY4741 STD1::TAP-HIS3MX6
CM003	*MATα* BY4741 URH1::TAP-HIS3MX6
CM005	BY4741 *MATa/MATα* URH1::TAP-HIS3MX6 NRK1::TAP-HIS3MX6
CM018	BY4741 *MATa/MATα* URH1::TAP-HIS3MX6 NPT1::TAP-HIS3MX6
CM019	BY4741 *MATa/MATα* URH1::TAP-HIS3MX6 SIR2::TAP-HIS3MX6
CM022	BY4741 *MATa/MATα* URH1::TAP-HIS3MX6 QNS1::TAP-HIS3MX6
CM023	BY4741 *MATa/MATα* URH1::TAP-HIS3MX6 UTR1::TAP-HIS3MX6
CM034	BY4741 *MATa/MATα* URH1::TAP-HIS3MX6 NMA1::TAP-HIS3MX6
CM035	BY4741 *MATa/MATα* URH1::TAP-HIS3MX6 NMA2::TAP-HIS3MX6
CM036	BY4741 *MATa/MATα* URH1::TAP-HIS3MX6 PNC1::TAP-HIS3MX6
CM041	BY4741 *MATa/MATα* URH1::TAP-HIS3MX6 ISN1::TAP-HIS3MX6
CM043	BY4741 *MATa/MATα* URH1::TAP-HIS3MX6 POS5::TAP-HIS3MX6
CM046	BY4741 *MATa/MATα* URH1::TAP-HIS3MX6 STD1::TAP-HIS3MX6

### Urh1 purification, specific activity assay and protein copy number determination

Recombinant Urh1 was expressed and purified as described [27]. To measure the NR hydrolytic activity of Urh1, we made use of the absorbance drop between NR and Nam at 269 nm, which is 2100 M^−1^ cm^−1^. In brief, 12 ng recombinant Urh1 or 30 µg yeast extract was incubated with 160 µM NR (10-fold higher than the *K_m_* value) in 50 mM Tris-HCl at pH 6.8 in a 3 mm path length cuvette [27]. Reactions were followed continuously in an Ultraspec 4000 UV/Vis spectrophotometer (Amersham Pharmacia Biotech, Freiburg, Germany) at 269 nm with SWIFT II version 2.03 software and were converted to Specific Activity (SA) in units of nmol/min/µg. To determine whether there is any NR hydrolysis activity in a *urh1* knockout strain, 0 to 12 ng of recombinant Urh1 protein plus or minus 30 µg yeast protein extract from strain CM001 was used to measure Urh1 SA. Protein copy number per cell was calculated from the ratio of the SA of Urh1 in the extract to that of pure Urh1, making use of 6 pg as the protein content per haploid yeast [28] and 37,900 Da as the molecular weight of Urh1 as in Formula 1:
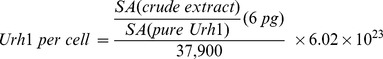



Protein copy number of Urh1 in a diploid strain heterozygous for tagged Urh1 was determined after demonstration that a wild-type diploid has the same SA as the strain heterozygous for the tag. Using 8 pg as the protein content per diploid cell [28] and 57,900 Da as the molecular weight of Urh1-TAP, Formula 2 was applied:
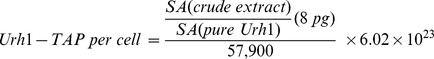



Copy numbers of all the other enzymes were obtained by multiplying Urh1-TAP copy number in a given condition by the ratio of TAP-tag western signals between the other enzymes and Urh1-TAP, *i.e.*, Pnc1-TAP/Urh1-TAP.

### Cell extract preparation and western blotting

A single colony was inoculated in 5 ml YP 2% glucose media and allowed to divide until OD_600 nm_ reached 0.5. Cells were inoculated into 30 ml YPD cultures with 2%, 0.5% or 0.2% glucose at initial OD_600 nm_ of 0.0005. Cells were pelleted when the OD_600 nm_ reached 0.5 and lysed by glass bead beating. For western blotting, GAPDH was the loading control and 20 µg total protein extract were loaded per lane on Biorad stain-free gels. TAP-tagged proteins were detected with anti-TAP tag antibody CAB1001 (Open Biosystems) as the primary reagent and horseradish peroxidase (HRP)-conjugated goat anti-rabbit antibody as the secondary reagent (Thermo Scientific). Sir2 protein was detected with anti-Sir2 antibody sc-6666 (Santa Cruz) as the primary reagent and HRP-conjugated donkey anti-goat antibody as the secondary (Abcam). GAPDH was detected with HRP-conjugated anti-GAPDH antibody (Abcam). Signals were visualized with SuperSignal West Femto Chemiluminescent substrate (Pierce), imaged with a ChemiDoc XRS^+^ system (Bio-Rad), and quantified with ImageLab software. All data were collected from three individual experiments and statistical data were analyzed by one-way ANOVA.

### Glucose concentration measurement

To measure the glucose concentration in yeast media, a single colony of CM018, CM019 and CM022 was inoculated in 5 ml YP 2% glucose media and allowed to grow until OD_600 nm_ reached 0.5. Cells were then inoculated into 50 ml YPD cultures with 2%, 0.5% or 0.2% glucose at an initial OD_600 nm_ of 0.0005. 0.2 ml media samples removed at indicated cell densities were centrifuged at 800 *g* for 10 min at 4°C to pellet yeast cells. Supernatants were then transferred to new tubes and stored at −20°C until measurement. Glucose concentration was assayed by using a Glucose Colorimetric Assay Kit (Cayman Chemical) per the manufacturer's protocol.

### Protein copy number determination in SDC media

See Methods S1

## Results

### Novel quantification of protein copy number in yeast

**Figure 2:**
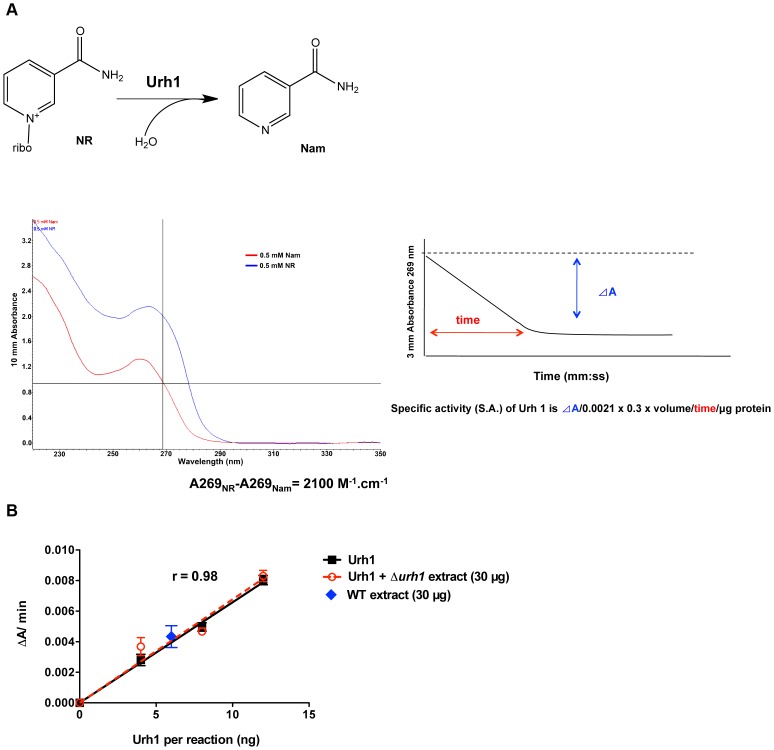
Urh1 enzyme activity in crude lysate can determine Urh1 absolute copy number. (A) Diagram of Urh1 SA measurements. Urh1 functions to hydrolyze NR to Nam. UV scanning result with 0.5 mM NR and Nam revealed a significant absorbance difference between NR and Nam at 269 nm, which is 2100 M^−1^cm^−1^. Urh1 SA can be calculated by monitoring the absorbance drop at 269 nm per time and protein used in the reaction. (B) Urh1 enzyme activity measurement was unaffected by addition of Urh1-free yeast extracts. Urh1 hydrolytic rate was measured with 0 to 12 ng of Urh1 in the absence or presence of 30 µg crude yeast extract from a *urh1* knockout strain. Correlation between the Urh1 and Urh1 plus *urh1*Δ extract group was 0.98.

At least 12 enzymes mediate the biosynthetic interconversion of NAD^+^ metabolites, which include two pyridine bases, two nucleosides, two mononucleotides, and five dinucleotides. Within the set of metabolic enzymes, Urh1 is an abundant enzyme responsible for converting NR to Nam plus ribose. Though two phosphorylases have the ability to convert NR to Nam plus a ribosyl product [20], [27], Urh1 is the only yeast enzyme with NR hydrolase activity, *i.e.*, the ability to convert NR to Nam in phosphate-free buffer. Taking advantage of this and with the aim of using Urh1 SA to determine its protein copy number, we developed a simple method to measure Urh1 SA by monitoring hydrolytic activity of NR. As shown in Figure 2A, NR and Nam exhibit a significant absorbance difference (2100 M^−1^ cm^−1^ at 269 nm), which was exploited to calculate Urh1 SA.

To rule out a Urh1-independent NR degradative activity in crude yeast extract, cell extracts from *urh1* knockout strain CM001 were used as control. As shown in Figure 2B, the hydrolytic rate of recombinant Urh1 was linear in the absence or presence of Δ*urh1* yeast extract. Interpolated from linear regression of recombinant Urh1 activity, there was about 6 ng of Urh1 protein in 30 µg of crude wild-type yeast extract. Because the amount of total protein per cell can be taken as a constant [28], the ratio of crude SA of Urh1 to the SA of purified Urh1 allows one to calculate Urh1 copy number per cell.

Given a facile method to determine the protein copy number of Urh1, we developed a second tool to obtain protein copy numbers of any other yeast protein. In brief, we established 11 yeast strains in which an in-frame tandem affinity protein (TAP)-tag was integrated at the C-terminus of Urh1 coding sequences and the same TAP-tag was integrated at the C-terminus of a second enzyme in NAD^+^ metabolism (Table 1). The resulting yeast strains were then grown in YPD at three concentrations of glucose. Extracted cellular proteins were analyzed by western blot using an anti-TAP tag antibody followed by detection with HRP-conjugated goat anti-rabbit antibody and chemiluminescent signal development. Chemiluminescent signals quantified by ImageLab software were used to determine the relative level of expression of each protein to Urh1-TAP under each experimental condition (Figures 1A, 1B and S3).

Because protein expression was expected to vary over a wide range, we aimed to determine whether this method is quantitatively sound. As shown in Figure 1C and Figure S1, the ratios between Urh1-TAP tagged protein and other tagged proteins were consistent in all serial dilutions of extract. These data indicate that the relative quantification method provides valid expression information over a wide range. Because each enzyme contains one TAP epitope and the SA of Urh1-TAP can be converted to its protein copy number per cell, the result of this analysis is a calculation of cellular protein copy number of each enzyme in each experimental condition.

### Sir2 and Pnc1 copy numbers increase during glucose restriction in rich media

**Figure 3:**
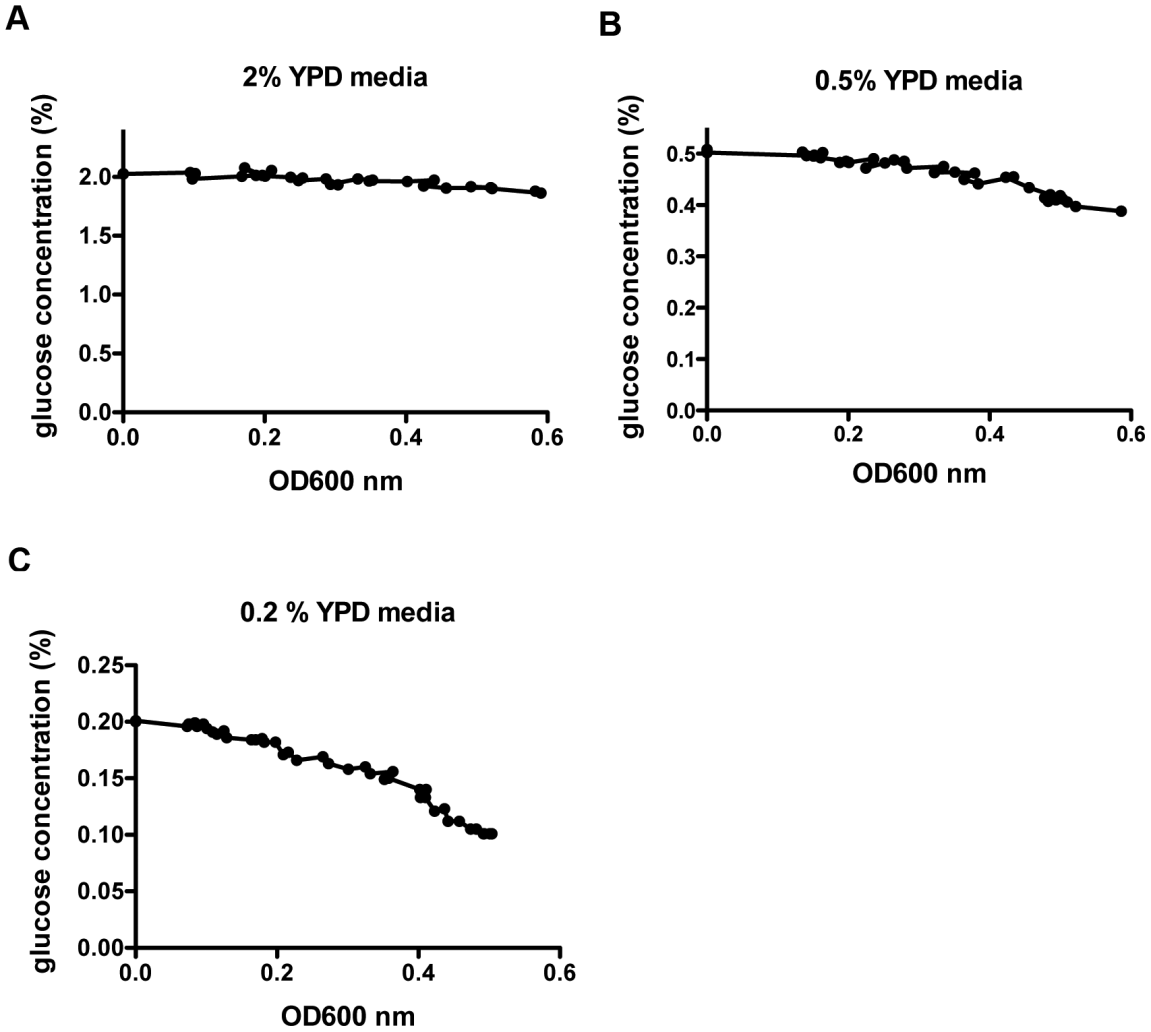
Glucose concentrations are maintained at acceptable levels up to OD_600 nm_  = 0.5. Glucose concentrations of media from yeast strains cultured in (A) 2.0% YPD media (B) 0.5% YPD media and (C) 0.2% YPD media were measured with Glucose Assay Kits (Cayman). Data were collected from three different diploid TAP-tag yeast strains and shown as mean ± SD.

After establishing methods to quantify protein copy numbers, we applied this technology to determine copy number changes of enzymes in NAD^+^ metabolism during glucose restriction under the conditions of most replicative longevity experiments, *i.e.* in rich YPD media. We aimed to maintain yeast cells in specific conditions for 10 generations to ensure evaluation of steady-state expression. However, because cell division consumes glucose, sample collection was optimized so that final glucose concentrations were maintained near the levels of initial innocula. As shown in Figure 3, with an initial OD_600 nm_ of 0.0005, a ten generation growth to an OD_600 nm_ of 0.5 produced only a drop of ∼ 0.1% glucose in each culture condition.

In all conditions, the ratio of crude Urh1 SA to that of homogeneous enzyme was consistently 1∶6200. These data indicate that there are ∼1.3 fg of Urh1-TAP per cell. Using the predicted molecular weight of the Urh1-TAP tagged construct of 57.9 kDa, this translates to an apparent Urh1 protein copy number of ∼14,000 in all conditions examined (Table S1).

In rich media conditions at 2% glucose, at a range of 38,000 to 45,000 molecules per cell, the most abundant NAD^+^ biosynthetic enzyme measured is Npt1, which converts NA to nicotinic acid mononucleotide (NAMN). Though Npt1 is crucial to maintain the level of NAD^+^ in yeast [29] and is required for the longevity benefit of CR [5], Npt1 is neither increased nor decreased in copy number by glucose restriction. The second most abundant enzyme in rich media, high glucose conditions is Pnc1, the nicotinamidase that converts Nam to NA. In 2% glucose, there are 18,000 molecules per cell. However, its copy number rises to 36,000 when glucose is reduced to 0.5% and to 49,000 when glucose is reduced to 0.2%. Increased expression of Pnc1 in stress and low glucose conditions has been previously reported and is due to increased mRNA expression driven by 5′ stress response elements [30]. Nma1, one of two mononucleotide adenylyltransferases [31], and Pos5 and Utr1, the two major NAD^+^/NADH kinases [18] are expressed at about 10,000 copies per cell and are unaffected by glucose concentrations. Nma2, the other mononucleotide adenylyltransferase [9] and the three enzymes that interconvert NR and nicotinic acid riboside (NAR) to nicotinamide mononucleotide (NMN) and NAMN, namely Nrk1 [16], Isn1 and Sdt1 [22] are expressed at 2,000 copies per cell under each condition. Though discovery of NR as a vitamin that can bypass *de novo* biosynthesis of NAD^+^ and NAD^+^ biosynthesis from conventional niacins [16] undermined the early annotations of glutamine-dependent NAD^+^ synthetase Qns1 as an essential gene, Qns1 can be considered to be essential unless NR is available. Despite this central role in NAD^+^ homeostasis, Qns1 has the lowest level of expression of any NAD^+^ metabolic enzyme examined in YPD at fewer than 2,000 copies per cell.

**Figure 4:**
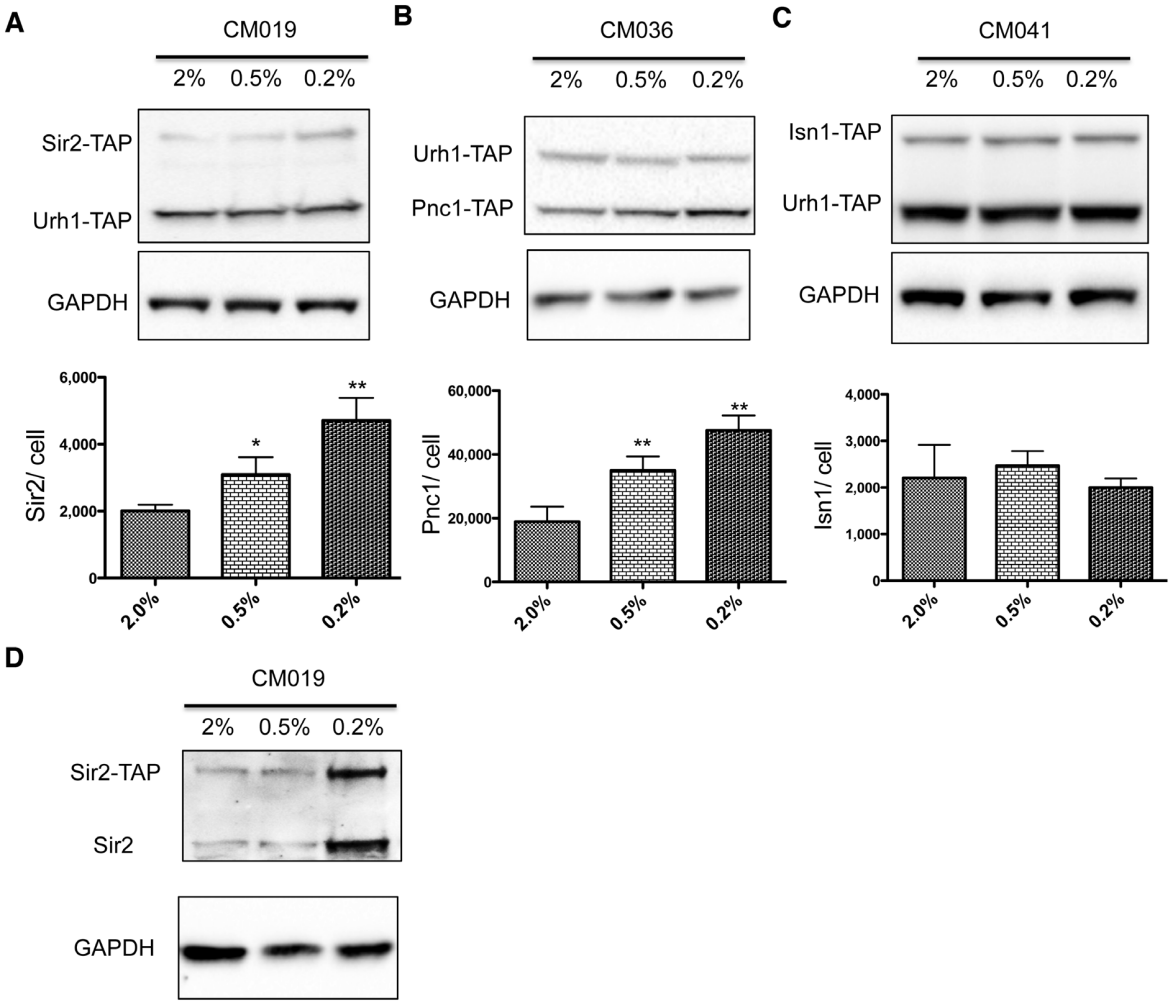
Pnc1 and Sir2 are induced by glucose restriction. Cell extracts from different dually TAP-tagged strains were prepared and analyzed by western blot. Urh1 expression data were converted to protein copy number using SA measurements as described in Materials and Methods. (A) Sir2 expression increases from 2,000/cell at 2% glucose to 4,900/cell at 0.2% glucose. (B) Pnc1 expression increases from 18,000/cell at 2% glucose to 49,000/cell at 0.2% glucose. (C) Isn1 expression is roughly constant at 2,000/cell under all conditions examined. (D) CM019 cell extracts probed with Sir2 antibody established no significant difference in protein expression and stability between endogenous and TAP-tagged Sir2 proteins.

**Figure 5:**
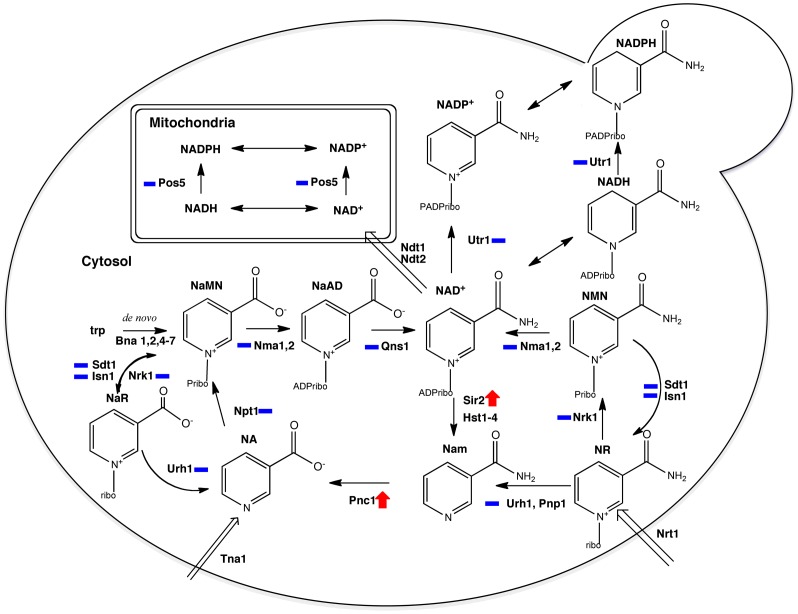
Summary of protein expression changes in CR. Overall, the abundance of most enzymes involved in NAD^+^ metabolic pathways was unchanged by CR. The only two enzymes with significant alterations were Sir2 and Pnc1, which are two consecutive enzymes that convert NAD^+^ into NA. Both are increased by CR in YPD. Pnc1 alone is increased in SDC.

Sir2, a protein lysine deacetylase that converts NAD^+^ to Nam [32], is required for longevity of long-lived Fob1-wild-type yeast strains [5], . Though much work has focused on potential mechanisms by which glucose levels might control Sir2 enzyme activity [5], [6], [9]–[13], here we show that the level of Sir2 protein is only 2,000 copies per cell at 2% glucose and increases to 3,200 and 4,900 copies per cell at 0.5% and 0.2% glucose, respectively, in YPD media. Though Sir2 expression and localization are altered by ribosomal DNA copy number [33], the level of expression of Sir2 as a function of glucose had been thought to be constant [34]. However, the lack of an earlier observation of the increase in Sir2 expression might be due to the short time of exposure to CR conditions [34]. In summary, among 12 NAD^+^ metabolic enzymes, Sir2 and Pnc1 are the only two enzymes affected by glucose restriction (Tables 2 and S1, Figures 4, 5 and S2) To test whether the changes were confined to the expression or stability of the TAP-tagged fusion protein, we prepared cell extracts from strain CM019 and probed with a Sir2 antibody. As shown in Figure 4D, the ratio between Sir2-TAP and un-tagged Sir2 protein was consistent in all three conditions, which indicated that quantification of TAP-tagged fusion protein reflects endogenous protein expression levels.

**Table 2:** Protein copy number of enzymes in the NAD^+^ metabolic pathway.

Protein Name	Culture Condition	Copy Number
**Isn1**	2.0%	2,200±700
	0.5%	2,500±300
	0.2%	2,000±200
**Nma1**	2.0%	10,000±1,000
	0.5%	10,000±1,000
	0.2%	9,400±1,000
**Nma2**	2.0%	2,000±200
	0.5%	2,300±300
	0.2%	2,000±100
**Npt1**	2.0%	45,000±4,000
	0.5%	42,000±7,000
	0.2%	38,000±8,000
**Nrk1**	2.0%	2,000±200
	0.5%	1,900±300
	0.2%	2,200±600
**Pnc1**	2.0%	18,000±4,000
	0.5%	36,000±3,000
	0.2%	49,000±4,000
**Pos5**	2.0%	9,200±1,200
	0.5%	9,500±1,700
	0.2%	11,000±2,000
**Qns1**	2.0%	1,500±200
	0.5%	1,800±500
	0.2%	1,800±300
**Sir2**	2.0%	2,000±200
	0.5%	3,200±500
	0.2%	4,900±700
**Std1**	2.0%	3,700±200
	0.5%	3,600±100
	0.2%	3,600±400
**Urh1**	2.0%	13,000±1,000
	0.5%	14,000±1,000
	0.2%	15,000±1,000
**Utr1**	2.0%	13,000±1,000
	0.5%	12,000±1,000
	0.2%	13,000±1,000

### Reduced expression of most NAD^+^ metabolic enzymes in synthetic media

Unlike YPD media, which contains multiple salvageable NAD^+^ metabolites, SDC media is restricted to tryptophan as a *de novo* NAD^+^ precursor and NA as a salvageable vitamin. As shown in Tables S2 and S3, most NAD^+^ enzymes are substantially reduced in protein accumulation in SDC media with respect to YPD. One notable difference between YPD expression and SDC expression was in accumulation of the two mononucleotide adenylyltransferases, Nma1 and Nma2. In YPD, Nma1 is the dominant enzyme at ∼10,000 copies per cell while Nma2 accumulates to about 20% of this copy number. However, in SDC, the Nma1 level decreased to about 500 copies per cell while Nma2 increased to about 2,500 copies per cell, effectively becoming the dominantly expressed enzyme in SDC.

As in YPD, the concentration of glucose had little effect on accumulation of the vast majority of NAD^+^ enzymes. Whereas Sir2 increased accumulation in glucose restricted YPD media, it did not do so in glucose restricted SDC media. However, Pnc1, which was not reduced in expression by SDC responded to glucose restriction with CR-induced protein expression.

## Discussion

The connection between NAD^+^ metabolism and lifespan extension by CR has been studied for more than a decade. Sir2 and Sir2 paralogs have been considered the major targets, which connect the requirement of NAD^+^ salvage to CR-induced lifespan extension [7], [9], [10], [12], [13], [35], [36]. Besides its function as limiting the production of extra-chromosomal ribosomal DNA circles (ERCs), Sir2 is also important for asymmetrical cell division [37]–[39], and for deacetylation of histone H4 Lys16, which is important for maintaining telomere function in older cells [40]. It has been shown in multiple studies that NAD^+^ levels in bulk cells are unchanged by CR [9], [12], [13], [24], [35]. In addition, having shown that intracellular NAD^+^ metabolites are not altered by CR [24], here we developed a method to quantify the copy number of enzymes involved in NAD^+^ metabolic pathways during normal and CR conditions. Our data indicate that levels of Sir2 and Pnc1 are increased by CR in YPD and that only Pnc1 is increased in expression by CR in SDC, while all other enzymes remain unchanged in protein expression. Though Sir2 and Pnc1 are capable of increasing degradation of NAD^+^ to NA, previous observations of the intracellular NA level indicate that it remains below 0.5 µM in all conditioned examined [24].

Another interpretation of increased expression of Pnc1 is that it reduces the concentration of Nam, a metabolite that inhibits Sir2 at high concentrations [13]. However, because addition of NA to synthetic media elevates the concentration of Nam by 15-fold, while glucose restriction in YPD elevates Nam by a further 50% [24], there is a lack of credible evidence that Nam at intracellular concentrations of up to 50 µM shortens lifespan. Increased Pnc1 expression might either be a noncausal epiphenomenon that correlates with CR-induced lifespan extension or it could increase the rate of production of NA in a manner or pathway that does not elevate intracellular NAD^+^ metabolites in bulk cells.

Here we showed that Urh1 copy number can be quantified in crude lysates by measurement of NR hydrolysis in phosphate-free buffer. By introducing TAP tags of Urh1 and any other protein into the same yeast strain, relative expression data obtained from western blotting were converted to protein copy number per cell. It is anticipated that this technology will be applied to diverse problems in yeast molecular and cellular biology.

## Supporting Information

Figure S1 **Western analysis of CM005 and CM034 strains.** Cell extracts from (A) CM005 (Urh1-TAP, Nrk1-TAP) and (B) CM034 (Urh1-TAP, Nma1-TAP) analyzed over a range of dilutions further establish the linearity of TAP-tagged detection.
(TIF)

Figure S2 **Western blot results of co-TAP-tagged strains.** Equal amounts of cellular extracts isolated from dually TAP-tagged strains cultured in 2%, 0.5% or 0.2% glucose YPD media were used to perform western blot with an anti-TAP antibody. All results showed no significant difference between each culture condition. (A) CM005; (B) CM018; (C) CM022; (D) CM023; (E) CM034; (F) CM035; (G) CM043; (H) CM046.
(TIF)

Figure S3 **GAPDH and stain-free gel images served as loading controls.** (A) 20 µg of cell extracts from dually TAP-tagged strains at 2% glucose were separated in 7.5% TGX stain-free gels and imaged with ImageLab software. (B) GAPDH signal detected with specific antibody was used as loading control.
(TIF)

Table S1 **Urh1 copy number in each dual-tag yeast strains in YPD media.**
(DOCX)

Table S2 **Protein copy number of enzymes in the NAD+ metabolic pathway in SDC media.**
(DOCX)

Table S3 **Urh1 copy number in each dual-tag yeast strains in SDC media.**
(DOCX)

Methods S1 **Supplementary materials and methods.**
(DOCX)
