# Supplementary figures and images for: Quantification of Protein Copy Number in Yeast: The NAD+ Metabolome
Source: PLoS One. 2014 Sep 4;9(9):e106496. doi: 10.1371/journal.pone.0106496 (PMC4154715; doi:10.1371/journal.pone.0106496)

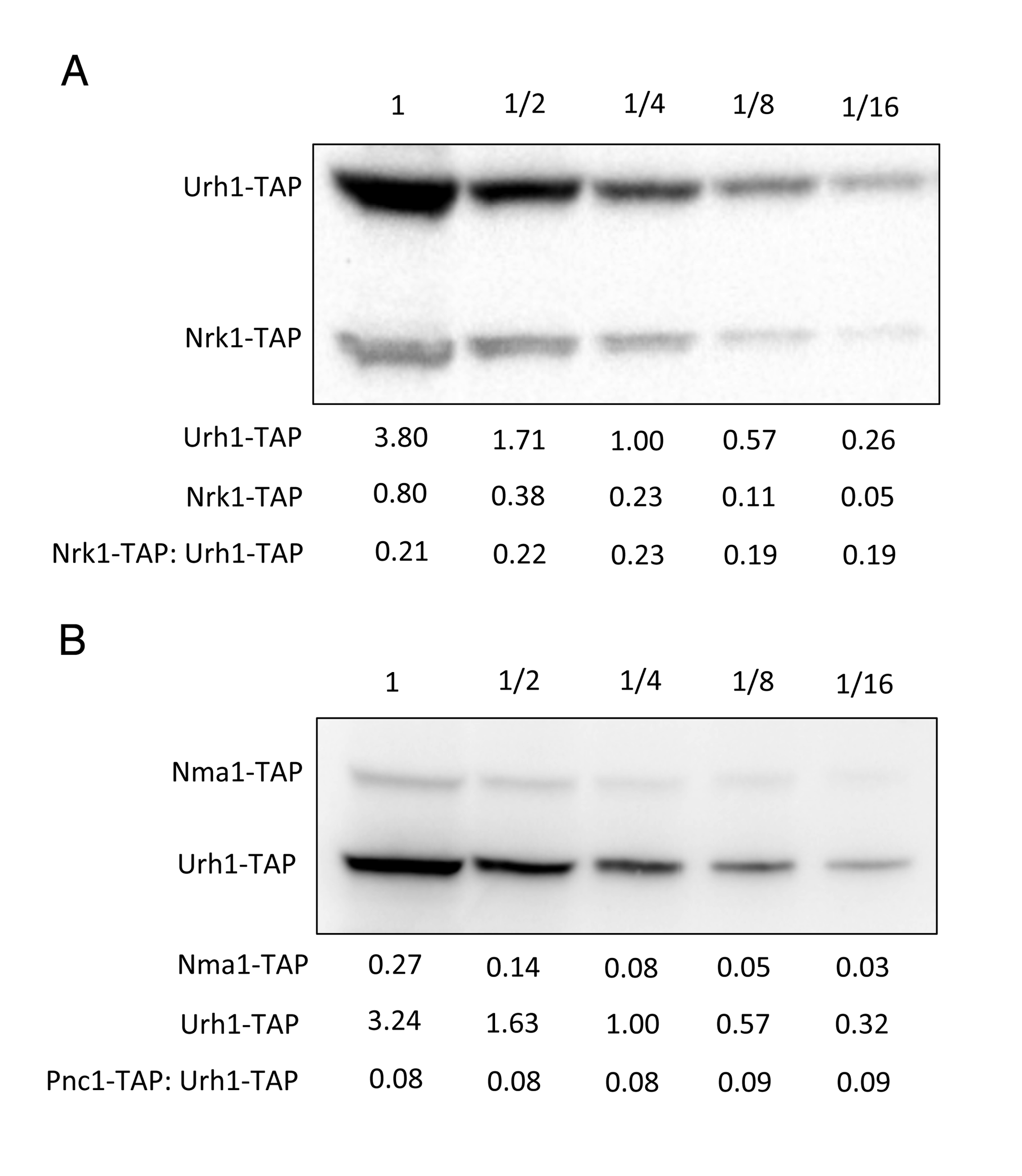

Supplement: Figure S1 — Western analysis of CM005 and CM034 strains. Cell extracts from (A) CM005 (Urh1-TAP, Nrk1-TAP) and (B) CM034 (Urh1-TAP, Nma1-TAP) analyzed over a range of dilutions further establish the linearity of TAP-tagged detection. (TIF) [file pone.0106496.s001.tif]

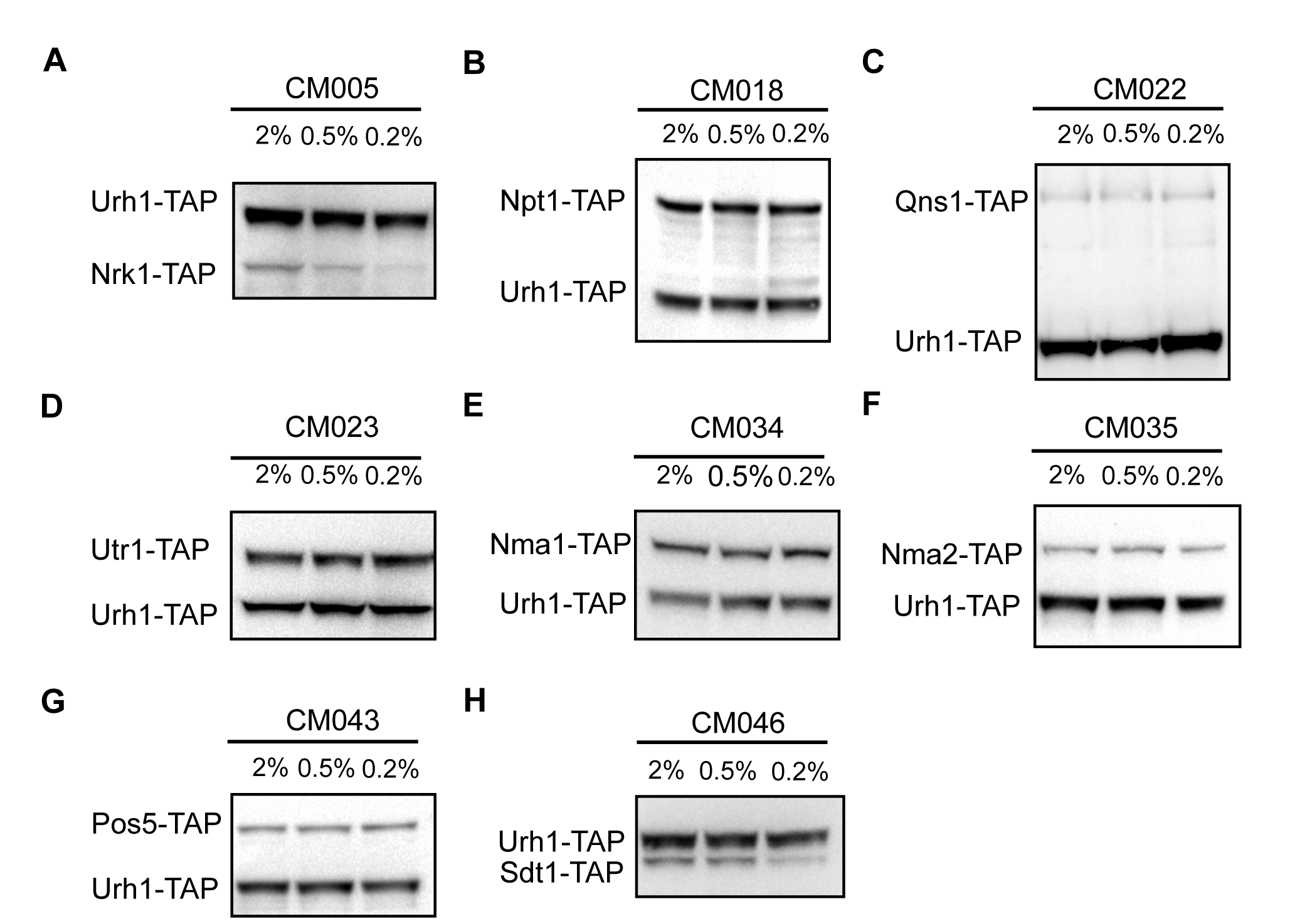

Supplement: Figure S2 — Western blot results of co-TAP-tagged strains. Equal amounts of cellular extracts isolated from dually TAP-tagged strains cultured in 2%, 0.5% or 0.2% glucose YPD media were used to perform western blot with an anti-TAP antibody. All results showed no significant difference between each culture condition. (A) CM005; (B) CM018; (C) CM022; (D) CM023; (E) CM034; (F) CM035; (G) CM043; (H) CM046. (TIF) [file pone.0106496.s002.tif]

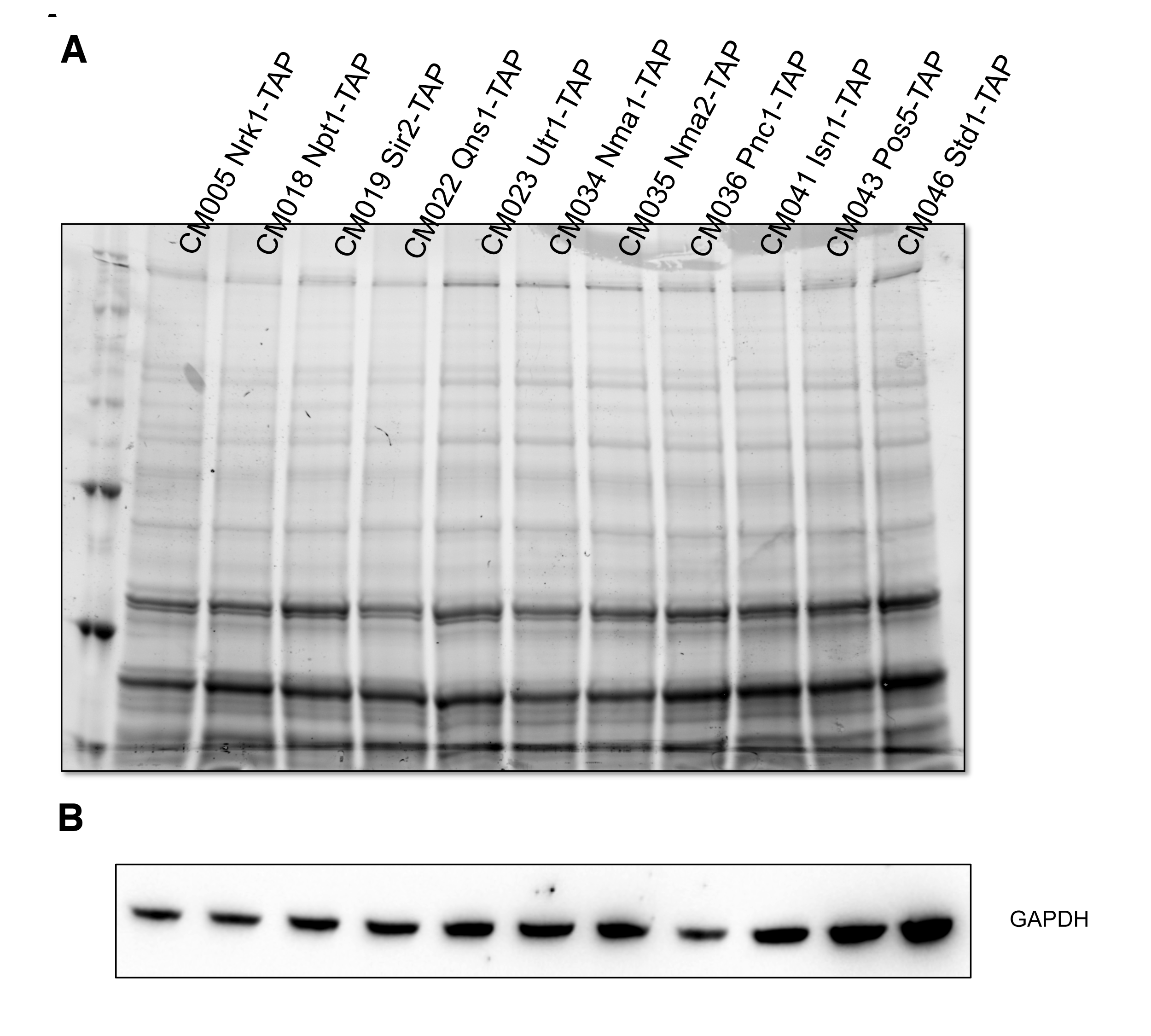

Supplement: Figure S3 — GAPDH and stain-free gel images served as loading controls. (A) 20 µg of cell extracts from dually TAP-tagged strains at 2% glucose were separated in 7.5% TGX stain-free gels and imaged with ImageLab software. (B) GAPDH signal detected with specific antibody was used as loading control. (TIF) [file pone.0106496.s003.tif]
