# Supplementary material for: Quantification of Protein Copy Number in Yeast: The NAD+ Metabolome
Source: PLoS One. 2014 Sep 4;9(9):e106496. doi: 10.1371/journal.pone.0106496 (PMC4154715; doi:10.1371/journal.pone.0106496)
Supplement: Table S1 — Urh1 copy number in each dual-tag yeast strains in YPD media. (DOCX) [file pone.0106496.s004.docx]

**Table S1** Urh1 copy number in each dual-tag yeast strains in YPD media

| Strain Name | Culture Condition | Copy Number |
| --- | --- | --- |
| CM005 | 2.0 % | 14,000 ± 1,000 |
|  | 0.5 % | 14,000 ± 1,000 |
|  | 0.2 % | 16,000 ± 1,000 |
| CM018 | 2.0 % | 13,000 ± 1,000 |
|  | 0.5 % | 14,000 ± 1,000 |
|  | 0.2 % | 14,000 ± 1,200 |
| CM019 | 2.0 % | 14,000 ± 1,200 |
|  | 0.5 % | 14,000 ± 1,000 |
|  | 0.2 % | 15,000 ± 1,000 |
| CM022 | 2.0 % | 13,000 ± 1,000 |
|  | 0.5 % | 14,000 ± 1,000 |
|  | 0.2 % | 15,000 ± 1,100 |
| CM023 | 2.0 % | 14,000 ± 1,000 |
|  | 0.5 % | 14,000 ± 1,000 |
|  | 0.2 % | 15,000 ± 1,100 |
| CM034 | 2.0 % | 13,000 ± 1,000 |
|  | 0.5 % | 14,000 ± 1,100 |
|  | 0.2 % | 14,000 ± 1,000 |
| CM035 | 2.0 % | 13,000 ± 1,000 |
|  | 0.5 % | 14,000 ± 1,300 |
|  | 0.2 % | 14,000 ± 1,100 |
| CM036 | 2.0 % | 13,000 ± 1,000 |
|  | 0.5 % | 14,000 ± 1,000 |
|  | 0.2 % | 15,000 ± 1,000 |
| CM041 | 2.0 % | 14,000 ± 1,300 |
|  | 0.5 % | 14,000 ± 1,100 |
|  | 0.2 % | 14,000 ± 1,100 |
| CM043 | 2.0 % | 13,000 ± 1,000 |
|  | 0.5 % | 13,000 ± 1,000 |
|  | 0.2 % | 15.000 ± 1,000 |
| CM046 | 2.0 % | 13,000 ± 1,000 |
|  | 0.5 % | 15,000 ± 1,000 |
|  | 0.2 % | 15,000 ± 1,000 |
