# Supplementary material for: Quantification of Protein Copy Number in Yeast: The NAD+ Metabolome
Source: PLoS One. 2014 Sep 4;9(9):e106496. doi: 10.1371/journal.pone.0106496 (PMC4154715; doi:10.1371/journal.pone.0106496)
Supplement: Table S2 — Protein copy number of enzymes in the NAD+ metabolic pathway in SDC media. (DOCX) [file pone.0106496.s005.docx]

**Table S2** Protein copy number of enzymes in the NAD^+^ metabolic pathway in SDC media

| Protein Name | Culture Condition | Copy Number |
| --- | --- | --- |
| Isn1 | 2.0 % | 1,600 ± 200 |
|  | 0.5 % | 1,700 ± 400 |
|  | 0.2 % | 1,900 ± 200 |
| Nma1 | 2.0 % | 510 ± 100 |
|  | 0.5 % | 500 ± 100 |
|  | 0.2 % | 490 ± 100 |
| Nma2 | 2.0 % | 2,600 ± 600 |
|  | 0.5 % | 3,100 ± 300 |
|  | 0.2 % | 2,800 ± 600 |
| Npt1 | 2.0 % | 23,000 ± 3,000 |
|  | 0.5 % | 27,000 ± 2,000 |
|  | 0.2 % | 25,000 ± 4,000 |
| Nrk1 | 2.0 % | 620 ± 100 |
|  | 0.5 % | 710 ± 100 |
|  | 0.2 % | 550 ± 100 |
| Pnc1 | 2.0 % | 17,000 ± 2,000 |
|  | 0.5 % | 24,000 ± 3,000 |
|  | 0.2 % | 48,000 ± 5,000 |
| Pos5 | 2.0 % | 6,600 ± 1,000 |
|  | 0.5 % | 6,600 ± 1,000 |
|  | 0.2 % | 6,900 ± 1,100 |
| Qns1 | 2.0 % | 840 ± 100 |
|  | 0.5 % | 730 ± 150 |
|  | 0.2 % | 820 ± 100 |
| Sir2 | 2.0 % | 370 ± 100 |
|  | 0.5 % | 320 ± 100 |
|  | 0.2 % | 310 ± 100 |
| Std1 | 2.0 % | 440 ± 100 |
|  | 0.5 % | 450 ± 100 |
|  | 0.2 % | 460 ± 100 |
| Urh1 | 2.0 % | 7,100 ± 1,200 |
|  | 0.5 % | 7,500 ± 1,000 |
|  | 0.2 % | 7,200 ± 1,000 |
| Utr1 | 2.0 % | 4,800 ± 1,000 |
|  | 0.5 % | 5,700 ± 1,000 |
|  | 0.2 % | 5,300 ± 1,000 |
