# Supplementary material for: Quantification of Protein Copy Number in Yeast: The NAD+ Metabolome
Source: PLoS One. 2014 Sep 4;9(9):e106496. doi: 10.1371/journal.pone.0106496 (PMC4154715; doi:10.1371/journal.pone.0106496)
Supplement: Table S3 — Urh1 copy number in each dual-tag yeast strains in SDC media. (DOCX) [file pone.0106496.s006.docx]

**Table S3** Urh1 copy number in each dual-tag yeast strains in SDC media

| Strain Name | Culture Condition | Copy Number |
| --- | --- | --- |
| CM005 | 2.0 % | 6,900 ± 1,000 |
|  | 0.5 % | 7,800 ± 1,000 |
|  | 0.2 % | 7,200 ± 1,000 |
| CM018 | 2.0 % | 6,300 ± 1,000 |
|  | 0.5 % | 7,500 ± 1,000 |
|  | 0.2 % | 6,600 ± 1,400 |
| CM019 | 2.0 % | 7,500 ± 1,100 |
|  | 0.5 % | 7,200 ± 1,000 |
|  | 0.2 % | 6,600 ± 1,100 |
| CM022 | 2.0 % | 7,500 ± 1,000 |
|  | 0.5 % | 6,900 ± 1,000 |
|  | 0.2 % | 7,500 ± 1,100 |
| CM023 | 2.0 % | 6,600 ± 1,000 |
|  | 0.5 % | 7,800 ± 1,000 |
|  | 0.2 % | 7,200 ± 1,000 |
| CM034 | 2.0 % | 7,200 ± 1,000 |
|  | 0.5 % | 7,200 ± 1,100 |
|  | 0.2 % | 7,200 ± 1,000 |
| CM035 | 2.0 % | 6,900 ± 1,000 |
|  | 0.5 % | 8,400 ± 1,000 |
|  | 0.2 % | 7,500 ± 1,000 |
| CM036 | 2.0 % | 6,900 ± 1,000 |
|  | 0.5 % | 7,800 ± 1,000 |
|  | 0.2 % | 7,500 ± 1,000 |
| CM041 | 2.0 % | 7,500 ± 1,000 |
|  | 0.5 % | 6,900 ± 1,000 |
|  | 0.2 % | 7,200 ± 1,300 |
| CM043 | 2.0 % | 7,200 ± 1,000 |
|  | 0.5 % | 6,600 ± 1,000 |
|  | 0.2 % | 7,500 ± 1,500 |
| CM046 | 2.0 % | 6,900 ± 1,100 |
|  | 0.5 % | 7,800 ± 1,000 |
|  | 0.2 % | 7,200 ± 1,200 |
