# Supplementary material for: Quantification of Protein Copy Number in Yeast: The NAD+ Metabolome
Source: PLoS One. 2014 Sep 4;9(9):e106496. doi: 10.1371/journal.pone.0106496 (PMC4154715; doi:10.1371/journal.pone.0106496)
Supplement: Methods S1 — Supplementary materials and methods. (DOCX) [file pone.0106496.s007.docx]

**Supplementary Materials and Methods**

Media

Media used for protein copy number analysis in SDC media (0.67 % Bacto-yeast nitrogen base without amino acids and 0.2% complete amino acid mix) was supplemented with filter-sterilized glucose at final concentrations of 2%, 0.5% or 0.2%.

Cell extract preparation

A single colony was inoculated in 5 ml SDC 2% glucose media and allowed to grown until OD_600 nm_ reached 0.5. Cells were inoculated into 30 ml SDC cultures with 2%, 0.5% or 0.2% glucose at initial OD_600 nm_ of 0.001. Cells were pelleted when the OD_600 nm_ reached 0.5 and lysed by glass bead beating. For Urh1 specific activity assay, 40 µg of cell extract were used in each reaction.
